# Structural and Pharmacological Network Analysis of miRNAs Involved in Acute Ischemic Stroke: A Systematic Review

**DOI:** 10.3390/ijms23094663

**Published:** 2022-04-23

**Authors:** Oscar Salvador Barrera-Vázquez, Juan Carlos Gomez-Verjan, Ricardo Ramírez-Aldana, Paola García-dela Torre, Nadia Alejandra Rivero-Segura

**Affiliations:** 1 Departamento de Farmacología, Facultad de Medicina, Universidad Nacional Autónoma de México, Mexico City 03500, Mexico; osbarrera6@gmail.com; 2 Dirección de Investigación, Instituto Nacional de Geriatría, Mexico City 10200, Mexico; jverjan@inger.gob.mx (J.C.G.-V.); rramirez@inger.gob.mx (R.R.-A.); 3 Unidad de Investigación Médica en Enfermedades Neurológicas, Centro Médico Nacional Siglo XXI, Instituto Mexicano del Seguro Social, Mexico City 06720, Mexico; pgarciatorre@gmail.com

**Keywords:** miRNAs, network biology, stroke, acute ischemic stroke, biomarkers, systematic review

## Abstract

Acute ischemic stroke (AIS) is among the main causes of mortality worldwide. A rapid and opportune diagnosis is crucial to improve a patient’s outcomes; despite the current advanced image technologies for diagnosis, their implementation is challenging. MicroRNAs have been recognized as useful as biomarkers since they are specific and stable for characterization of AIS. However, there is still a lack of consensus over the primary miRNAs implicated in AIS. Here, we performed a systematic review of the literature covering from 2015–2021 regarding miRNAs expression during AIS and built structural networks to analyze and identify the most common miRNAs expressed during AIS and shared pathways, genes, and compounds that seem to influence their expression. We identified two sets of miRNAs: on one side, a set that was independent of geographical location and tissue (miR-124, miR-107, miR-221, miR-223, miR-140, miR-151a, miR-181a, miR-320b, and miR-484); and on the other side, a set that was connected (hubs) in biological networks (miR-27b-3p, miR-26b-5p, miR-124-3p, miR-570-3p, miR-19a-3p, miR-101-3p and miR-25-3p), which altered *FOXO3*, *FOXO4*, and *EP300* genes. Interestingly, such genes are involved in cell death, FOXO-mediated transcription, and brain-derived neurotrophic factor signaling pathways. Finally, our pharmacological network analysis depicted a set of toxicants and drugs related to AIS for the first time.

## 1. Introduction

Acute ischemic stroke (AIS) is a significant public health problem, representing 80 to 90% of worldwide stroke cases [1]. The World Health Organization (WHO) and the Institute of Health Metrics and Evaluation stated that AIS is the second cause of morbidity, disability, and mortality in individuals over 60 years old [2]. AIS results from permanent local blockage of the arteries that supply glucose and oxygen to the brain. It requires rapid evaluation and treatment to achieve better outcomes [3,4]. Both endovascular and thrombolytic (recombinant tissue plasminogen activator, rtPA) therapies help restore cerebral blood flow. However, rtPA administration induces symptomatic intracerebral hemorrhage in 3% of AIS patients [5]; thus, clinicians are cautious with its administration. Therefore, AIS diagnosis is challenging since it can be confused with other types of stroke. Additionally, several factors, such as deficits in an accurate triage in emergency rooms, expenses, availability of experts, neuroimaging equipment, and a scarce education among the general population to identify a stroke, narrow the window of time to manage AIS [6,7] adequately. Hence, a more efficient, non-invasive, cheap, and sensitive strategy for proper AIS diagnosis is needed.

On this matter, several studies have focused on characterizing biomarkers to accurately differentiate among the most common stroke subtypes. Non-coding RNAs, such as microRNAs (miRNAs, small single-stranded non-coding RNA molecules from ~22 endogenously expressed nucleotides that can regulate gene expression through different epigenetic mechanisms), have been proposed as novel biomarkers. Since they are highly stable and differentially expressed in specific conditions, such as cancer, arthritis, osteoporosis, infectious diseases, cardiovascular diseases, neurodegenerative diseases, and AIS [8,9,10], miRNAs can be easily isolated from different liquid biopsies, such as whole blood, plasma, serum, blood circulating exosomes, peripheral blood cells, and cerebrospinal fluid, with low invasiveness. Additionally, they can easily be measured in conventional labs with the minimum requirements of molecular biology [11]. Notably, research in AIS has focused on characterizing miRNA profiles to accurately differentiate it from other stroke types [11,12,13,14,15,16,17]. However, a consensus on what is included in a miRNA-based biomarker panel for AIS diagnosis is still lacking.

Recently, biological networks have become relevant in biomedicine since they represent a holistic approach to associating and integrating experimental and epidemiological data, helping to fill the gap in a wide range of biological features occurring in a specific moment (or condition). Therefore, the use of these tools has become an emerging area, enabling an understanding of the interaction between biomarkers and therapeutic target discovery. In addition, current technologies provide large-scale biomedical datasets that can be used to understand the genesis of disease [18,19].

In this context, many studies have taken advantage of these tools and suggested a panel of miRNAs for AIS diagnosis [20,21,22,23,24]. Nevertheless, there is a lack of consensus on the miRNAs expressed in all of them. Such an issue limits the translation of these miRNAs into clinic practice. Therefore, in the present study, using a systems biology approach, we aimed to identify and propose a set of miRNAs, genes, pathways, and compounds involved in AIS to contribute to a better understanding of the regulation of miRNAs during stroke.

Hence, taking advantage of the studies regarding miRNAs and the differential expression of miRNAs in AIS, we performed a systematic review of the literature on miRNAs differentially expressed in AIS, published from 2015 to 2021. Once we curated the database, we performed structural network analysis to obtain the most connected nodes based on the origin of the biological samples and the geographical region. We identified the genes, pathways, and compounds (toxicants or drugs) involved in regulating such miRNAs with the most connected nodes.

## 2. Materials and Methods

### 2.1. Study Strategy and Selection

**Figure 1:**
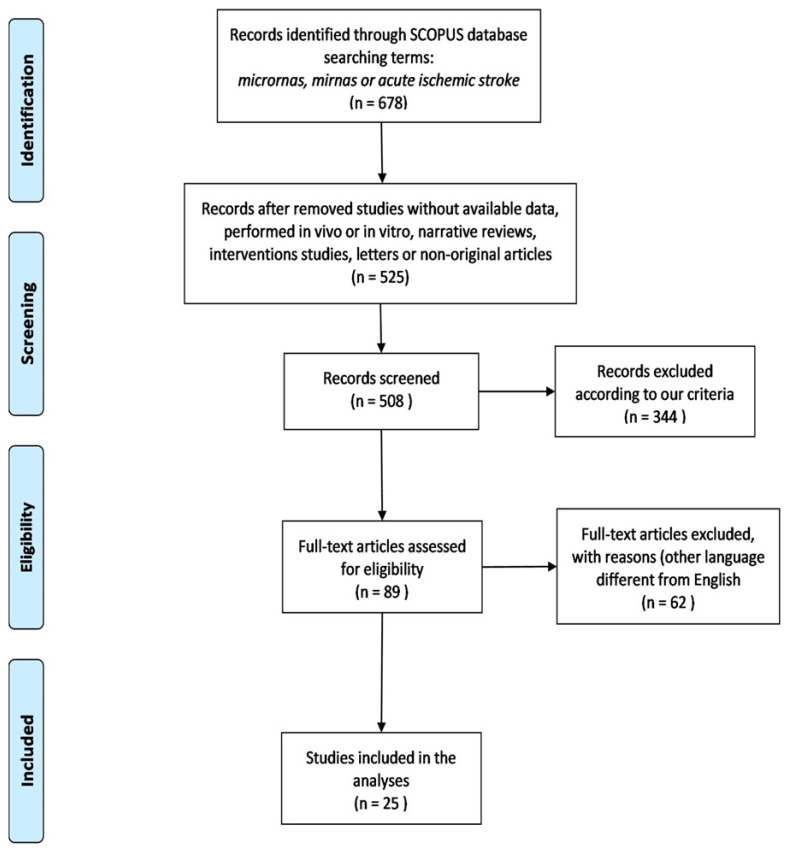
Flow diagram of the article selection for the meta-analysis, modified from Moher et al., 2009.

We followed the PRISMA statement (Moher et al., 2009) to perform this study (Figure 1). Methods were submitted to the PROSPERO database with the registration number **RD42020206145**. We used the MESH terms: microRNAs, miRNA, acute ischemic stroke, and brain stroke. Two independent reviewers searched all relevant studies from 2015 to 2021 in the Scopus database 2021.

Selected studies fulfilled the following eligibility criteria:

#### 2.1.1. Inclusion Criteria

1. Studies were reported or published between 2015 and 2021.
2. Studies discussed miRNAs differentially expressed in AIS.
3. Studies presented both cases and control groups.
4. Studies performed in human samples, such as whole blood, serum, plasma, exosomes, or blood cells; studies based on the ethnicity of study participants were not excluded.
5. Only studies that validated AIS diagnosis by neuroimaging, such as computed tomography (CT) or magnetic resonance imaging (MRI), were included.
6. Studies were conducted within 24 h of AIS symptoms.

#### 2.1.2. Exclusion Criteria

1. Studies published in languages excluding English.
2. Narrative reviews, intervention studies, letters to editors, and non-original articles.
3. Unpublished data, incomplete datasets, or preprints.
4. Studies without available data.
5. Studies without controls.
6. Studies that used duplicated data.
7. Studies performed in vivo.
8. Studies performed in vitro, even when these were human-derived.
9. Studies performed with already published databases.

### 2.2. Data Extraction

We retrieved the relevant information from each selected study, as depicted in Table S1 in the Supplementary materials.

#### Data Collection

The genes modulated by the miRNAs from the data extraction were searched for in the Target Expression Analysis section of the miRDB-MicroRNA Target Prediction Database (http://www.mirdb.org/mirdb/index.html/ accessed 1st December 2021). Only targets with a score >95% were considered for this study. The drugs responsible for the modulation of miRNAs involved in AIS were searched for in the mirNET database (https://www.mirnet.ca 29 December 2021) for the network analysis. The bar plot was generated using the ggplot2 package available in R using the information obtained in the Target Expression Analysis section of the miRDB-MicroRNA Target Prediction Database (http://www.mirdb.org/mirdb/index.html/ accessed 29 December 2021).

### 2.3. Network Structural Analysis

Networks were used to establish the most connected miRNAs and the most relevant pathways within AIS. They were built using Cytoscape software v 3.8 [25]. The most connected genes in the network were identified using the Cytohubba plugin [26]. Additionally, we used the BinGO plugin of Cytoscape [27] to identify the principal signaling pathways altered by the miRNAs. The most significantly enriched pathways (*p* < 0.05, *p*-values) were corrected using the Benjamin–Hochberg procedure, according to previous reports [28].

### 2.4. Network Pharmacology Analysis 

Structural networks were built using the Cytoscape software v 3.8 [25], and the chemical compounds responsible for the modulation of miRNAs involved in AIS were searched for in the mirNET database (https://www.mirnet.ca 29 December 2021). The most connected genes in the network were identified using the Cytohubba plugin [26]. Additionally, the most connected chemical compounds were determined according to their score, and their functions were investigated in the literature.

## 3. Results

According to our criteria, we identified 678 different studies by applying the filters mentioned in the experimental procedure section. From these, we used 25 studies to construct the networks. According to the PRISMA statement, Figure 1 depicts the article selection criteria for the subsequent analyses.

**Figure 2:**
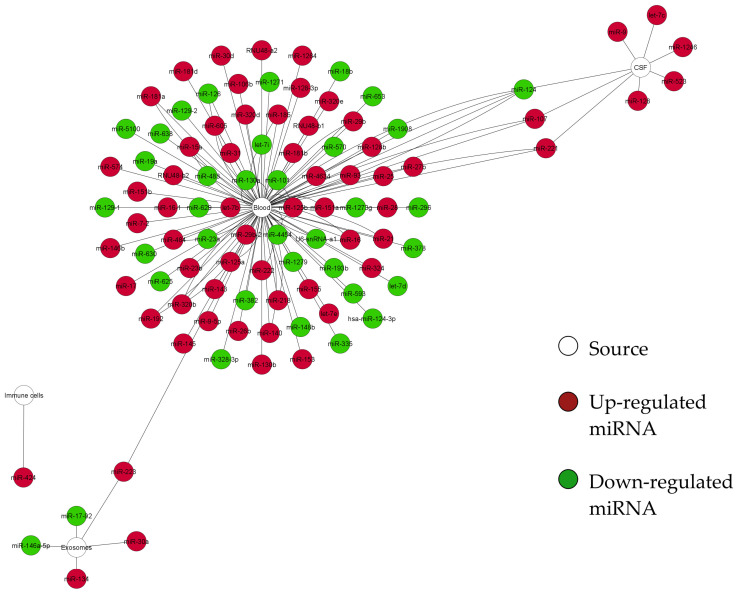
Structural network of the DE miRNAs derived from AIS patients according to the sample origin. The network represents miRNAs (nodes green or red) organized according to the source (white nodes) from which the samples were derived (Table S1). miRNAs appear as nodes colored in red (upregulated) or green (downregulated) according to their expression; edges indicate the number of independent studies reporting them. Four miRNAs are common in at least two different tissues (miR-124, miR-107, miR-221, and miR-223). Number of nodes: 103, number of edges: 133, network diameter: 6, network centralization: 0.887. The network was built using Cytoscape software (v.3.8.0). For a better image resolution, please review the Supplementary Materials (Figure S2).

**Figure 3:**
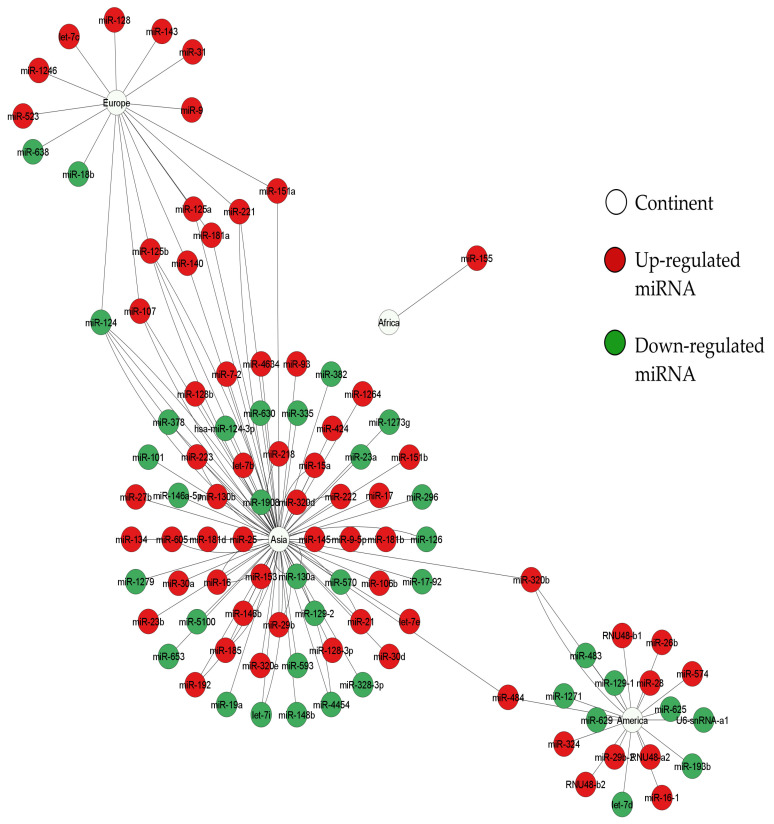
Structural network analysis of miRNAs that are differentially expressed in AIS from geographical samples. Data were extracted from 25 selected articles agreeing with our inclusion criteria (Table S1). Nodes correspond to miRNAs (targets according to their expression, those appear colored in red (upregulated) or green (downregulated)) and continents are shown as white nodes. Edges indicate different studies reporting the same miRNA from the same continent. (number of nodes: 103, number of edges: 133, network diameter: 6, network centralization: 0.713). The network was built using Cytoscape software (v.3.8.0). For a better image resolution, please review the Supplementary Materials (Figure S3).

We obtained a curated database of the differentially expressed miRNAs in AIS, and built a structural network analysis based on the origin of the sample (Figure 2). This network shows four miRNAs shared between blood and CFS (miR-124, miR-107, and miR-221) or between blood and exosomes (miR-223). Additionally, this network shows that blood is the tissue that shares most targets. Thus, it could be considered a hub in the network. Furthermore, we built another network to identify which miRNAs were shared between the geographical regions where the samples were collected. We grouped the samples according to the continent from which the samples came (Asia, Europe, Africa, and America. The latter refers only to studies performed in the USA since there is a lack of studies conducted in Latin America. As a result, using the network shown in Figure 3, we identified eight miRNAs (miR-124, miR-107, miR-221, miR-140, miR-151a, miR-181a, miR-320b, and miR-484) shared among Asia, Europe, and America while Africa stood alone from the network with miR-155.

**Figure 4:**
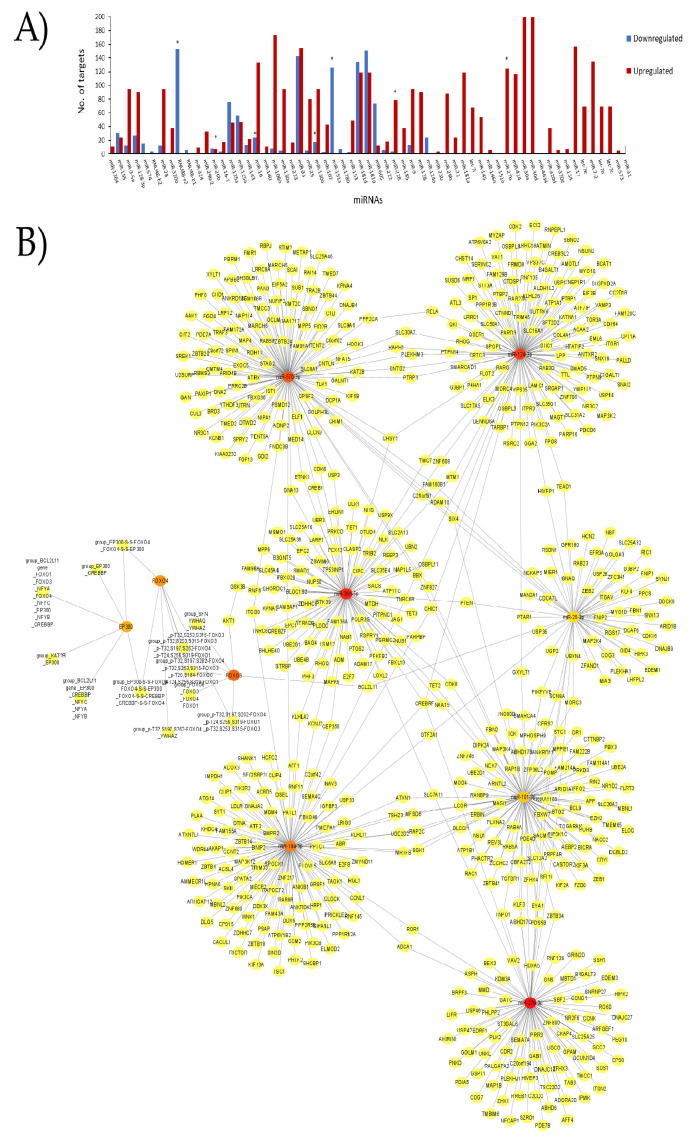
Analysis of miRNAs involved in AIS and their predicted targets. (**A**) The bar plot shows the number of targetable genes included in the miRNAs curated dataset. Bars colored in red show upregulated genes while bars in blue show downregulated genes. (**B**) The structural network was built with the Cytohubba plug-in for the top 10 most connected nodes (miRNAs and targets), including miR-27b-3p, miR-26b-5p, miR-124-3p, miR-570-3p, *FOXO3*, miR-19a-3p, *FOXO4*, *EP300*, miR-101-3p, and miR-25-3p. Nodes are colored according to the number of degrees and correspond to the most connected genes and miRNAs from the network (B). For a better image resolution, please review the Supplementary Materials (Figure S4).

Next, we used our complete dataset of miRNAs (Table S1) in the miRBD database (http://www.mirdb.org/mirdb/index.html/ accessed 29 December 2021) to identify genes and pathways that interact with them. We only considered target genes with a score above 95% (Figure 4). Figure 4A depicts the number of nodes (genes) that interact with the whole miRNA dataset. The most regulated targets as indicated by the miRBD database include miR-30a and miR-30d (200 targets), miR-106b (173 targets), miR-17 (156 targets), miR-93(154 targets), miR-124-3p (153 targets), miR-23a (151 targets), miR-126 (143 targets), and miR-7-2 (135 targets). With this new set, we built another network with the genes altered by these miRNAs. After performing network analysis, we identified the most connected nodes: miR-27b-3p, miR-26b-5p, miR-124-3p, miR-570-3p, *FOXO3*, miR-19a-3p, *FOXO4*, *EP300*, miR-101-3p, and miR-25-3p (Figure 4B).

Interestingly, these miRNAs are different from those first identified in Figure 2 and Figure 3, suggesting that despite a set of miRNAs sharing ubiquity in AIS, other miRNAs play a key role during AIS due to the number of genes for regulation. *FOXO3, FOXO4*, and *EP300* are involved in FOXO-mediated transcription of cell death genes, FOXO-mediated transcription, and brain-derived neurotrophic factor (BDNF) signaling. Additionally, we performed a gene enrichment analysis using our miRNA dataset with genes. We found that the most affected genes by our miRNA dataset are involved in the neurotrophin signaling pathway; cell cycle; cell death; the IL-2, IL-4, and IL-6 pathways; the leukocyte intrinsic hippo pathway; NAD metabolism; the endothelin pathway; cell adhesion; protein folding; apoptosis; angiogenesis; aging; oxidative stress; and mitochondrial membrane organization signaling, among others.

**Figure 5:**
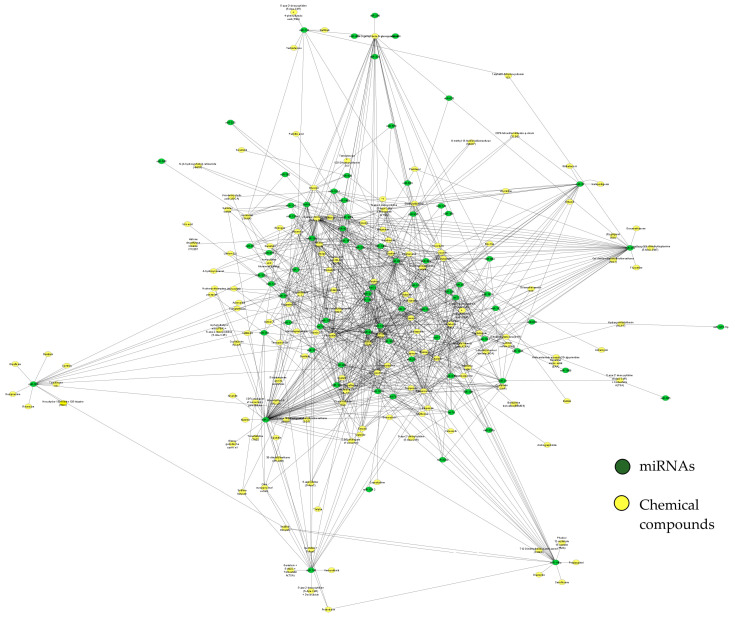
Structural network of drugs and their targeted miRNAs generated with miRNAs and their targets involved in AIS. This network addresses chemical compounds, such as drugs or xenobiotics, among others (shown as yellow nodes), associated with our miRNA dataset (green nodes) within the mirNET database (https://www.mirnet.ca/upload/MirUploadView.xhtml 29 December 2021). For a better image resolution, please review the Supplementary Materials (Figure S5).

Finally, we performed pharmacology network analysis on the related chemical compounds with our miRNA dataset within the mirNET database (https://www.mirnet.ca/upload/MirUploadView.xhtml 29 December 2021) (Figure 5 and Table 1). Interestingly, we identified drugs and toxicants that could modulate such miRNAs and, therefore, must be considered for toxicological analysis and further ecological and epidemiological studies.

## 4. Discussion

Research on biomarkers, particularly those based on miRNA profiles, seems valuable for the development of a reliable panel to distinguish between stroke subtypes [21,36,37]. As mentioned above, miRNAs can characterize several diseases, including stroke. In this context, several reports have suggested that miRNAs may be helpful in differentiating between ischemic stroke and hemorrhagic stroke. In this context, a set of miRNAs specific for the two major subtypes of hemorrhagic stroke have been reported: intracerebral hemorrhage (miR-130a, miR-29c, and miR-122) and subarachnoid hemorrhage (miR-132 and miR-324) [38]. Interestingly, these miRNAs are different from the miRNAs that our study identified for characterization of AIS (miR-124, miR-107, miR-221, miR-140, miR-151a, miR-181a, miR-320b, miR-484, miR-27b-3p, miR-26b-5p, miR-124-3p, miR-570-3p, miR-19a-3p, miR-101-3p, and miR-25-3p). On the other hand, miRNAs have been recognized as potential biomarkers since they are specific, sensitive, and represent a relatively simple method, which is non-invasive and currently affordable in most laboratories worldwide. In contrast, neuroimaging (CT or MRI) is expensive, requires highly trained personnel, and its availability in public health units is limited [39]. Moreover, the images obtained from the early stages of AIS are often mistaken for typical brain morphology. Large infarcts are only visible within 6 h after AIS [6], increasing the risk of developing an adverse prognosis for patients.

At first sight, our results indicate a potential set of miRNAs that may be used for AIS characterization. These miRNAs are involved in biological processes, such as excitotoxicity, neuronal death, inflammation, neurogenesis, and angiogenesis, which are all common mechanisms during AIS [40]. These miRNAs seem to be expressed independently from the tissues or the geographical origin of the samples. However, it is essential to highlight that our study also identified a lack of studies focused on the characterization of miRNAs expressed during AIS in the Latin American population, suggesting that for miRNAs to be translated into clinical practice, studies performed on this population are urgently required. Our results also demonstrate that most studies were performed in the blood (whole blood and derivates), which was reported as an enriched source of miRNAs [40,41,42], suggesting that further studies should continue to isolate miRNAs from blood.

Through network analysis, we found that the most connected genes altered by our miRNA dataset were *FOXO3, FOXO4*, and *EP300*. These targets have some critical roles within AIS. For instance, FOXOs transcription factors have critical roles in several processes, such as proliferation, apoptosis, autophagy, metabolism, inflammation, differentiation, and stress resistance [43]. It has been documented that FOXOs are involved in the injury following cerebral ischemia and play an essential role in cell death mechanisms [44,45]. Evidence has also shown that activated *FOXO3* plays a part in regulating autophagy in the brain, reducing the injury caused by cerebral ischemia-reperfusion, thus providing a new approach for further prevention and treatment of cerebral ischemia [46]. Besides, knockdown of *FOXO4* promotes cell proliferation, and inhibits cellular apoptosis via a reduction in oxidative stress after cerebral ischemia/reperfusion (CIR) injury, indicating that this could represent a new therapeutic target for the treatment of CIR injury [47]. *EP300*, on the other hand, has been associated with many different transcription factors involved in numerous cellular processes, such as growth, survival, apoptosis, and DNA repair [48].

Further, *EP300* acetylates members of the FOXO family, such as *FOXO1*, and enhances its transcriptional activity [49]. It has been proposed that CBP/p300 functions as a co-factor in FOXO-mediated transcriptional activity, whereas FOXO acetylation attenuates FOXO-mediated transcription of target genes [50]. Together, these data indicate a potential target for further interventions to prevent neuronal death during AIS.

On the other hand, for the first time, our study also identified compounds (toxicants and drugs) that regulate the expression of miRNAs involved in AIS. Such compounds are standard air or water pollutants, and others are therapeutic drugs [31,37]. These data open a novel line of study related to understanding the role of these compounds in the genesis of AIS or research on the neuroprotective compounds that protect against AIS. Thus, further pharmaco-epidemiological studies investigating the roles of these compounds in the development, prevention, and alleviation of AIS are highly required.

## 5. Conclusions

Our study has some limitations, such as the number of studies included, which corresponds to the heterogeneity and lack of relevant clinical data, including the lack of a complete survey about individuals’ lifestyles, and the heterogeneity in the protocols, sample collection, miRNA isolation methodologies, and platforms used for miRNA analysis. Despite such limitations, our study systematically recapitulated the most outstanding reports identifying the miRNA profiles exhibited during AIS and for the first time, we identified compound toxicants or drugs, such as arsenic, trichostatin, and 5-Aza-CdR that are related to AIS. Such compounds should be considered in further epidemiological studies to understand their potential as treatments for AIS or potential risk factors for the development of AIS. Additionally, since we identified a lack of Latin American profiles in AIS, we suggest that this population is considered and incorporated in further studies.

## Figures and Tables

**Table 1:** The most outstanding repositioned drugs lead to miRNAs involved in AIS from the pharmacology network analysis (the score is depicted as a node with more neighbors).

Name	Score	Function	Type of Agent
5-aza-2-deoxycytidine (5-Aza-CdR)	55	DNA methyltransferase inhibitor was able to reactivate genes silenced by DNA methylation and is a very potent epigenetic drug in several hematological malignancies [29].	Chemotherapeutic drug
5-Fluorouracil	42	In vitro studies have shown potential anticancer activity [30].	Chemotherapeutic drug
Ginsenoside Rh2	38	Major bioactive ginsenosides from *Panax ginseng* with anti-proliferation, anti-invasion, anti-metastasis, induction of cell cycle arrest, promotion of differentiation, and reversal of multi-drug resistance activities against multiple tumor cells, and also alleviates the side effects of chemotherapy or radiotherapy [31].	Chemotherapeutic drug
Formaldehyde	38	Formaldehyde alters miRNA patterns that regulate gene expression, potentially leading to the initiation of various diseases [32].	Carcinogenic compound
Arsenic trioxide	32	Alters the miRNA gene expression pattern in acute promyelocytic leukemia cells [33].	Toxicant
Trichostatin A (TSA)	29	A fungistatic antibiotic was obtained from *Streptomyces platensis*. It causes an accumulation of acetylated histones in a variety of mammalian tumor cell lines [34].	Antibiotic drug
1,2,6-Tri-O-galloyl-beta-D-glucopyranose	29	The natural compound from *Camellia sinensis* plays multiple roles against multidrug-resistant bacteria and other diseases [35].	Antibiotic drug

## Supplementary Materials

The following supporting information can be downloaded at: https://www.mdpi.com/article/10.3390/ijms23094663/s1.
