# Supplementary figures and images for: Structural and Pharmacological Network Analysis of miRNAs Involved in Acute Ischemic Stroke: A Systematic Review
Source: Int J Mol Sci. 2022 Apr 23;23(9):4663. doi: 10.3390/ijms23094663 (PMC9105699; doi:10.3390/ijms23094663)

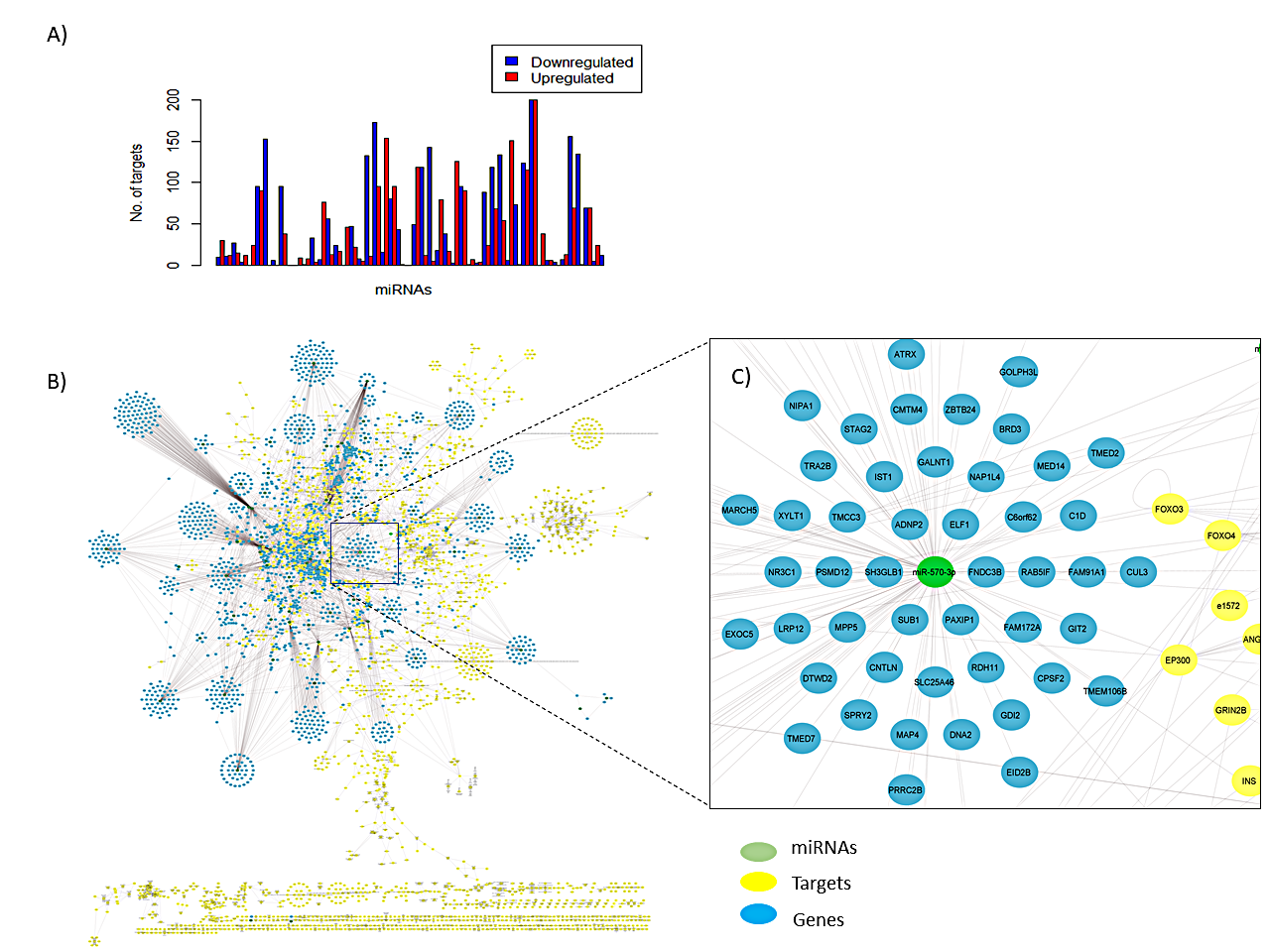

Supplement: Supplementary file 1 [file ijms-23-04663-s001.zip › ijms-1639567supp/Supplementary Files Figures/Figure 4S-2.tif]

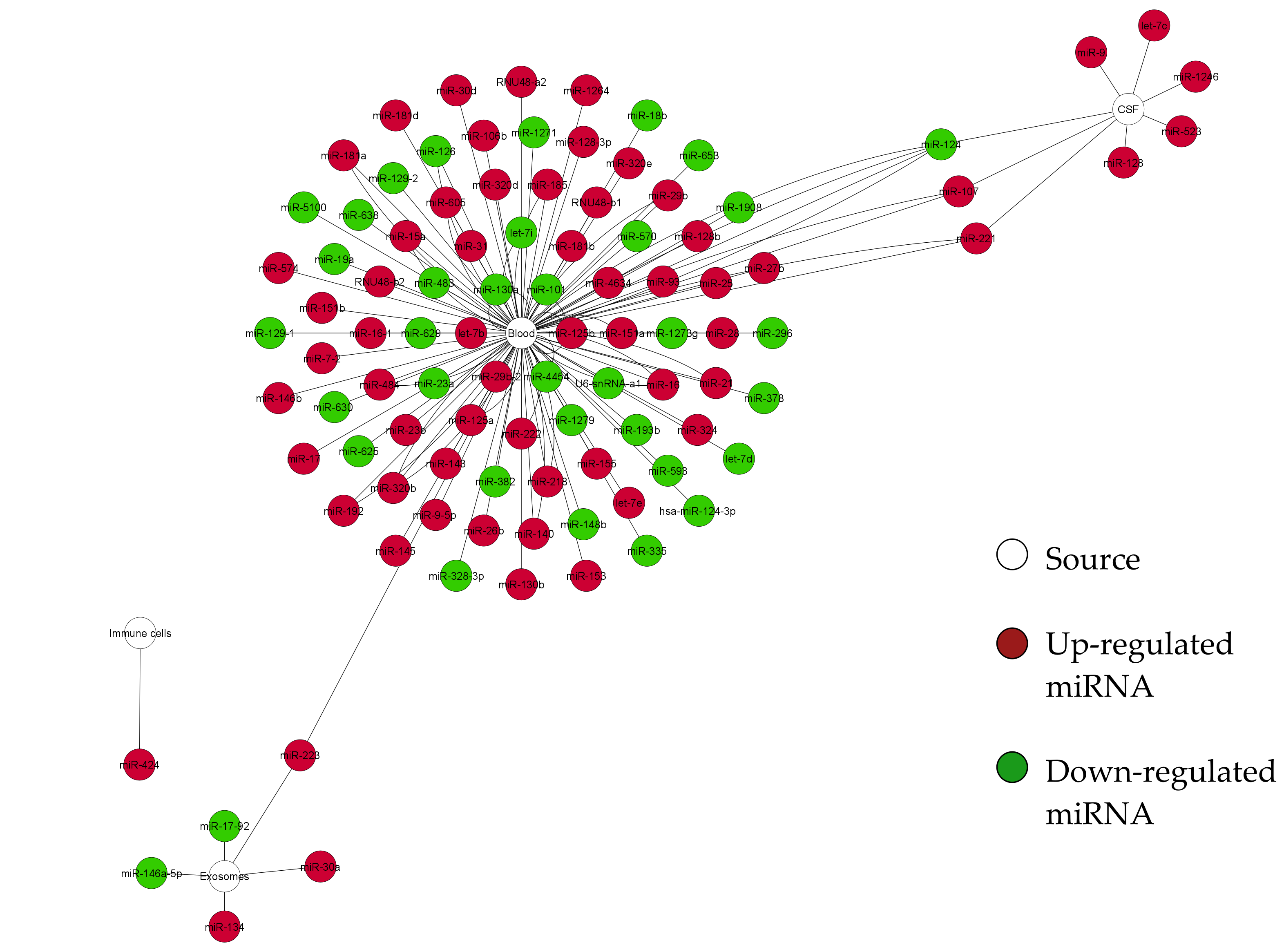

Supplement: Supplementary file 1 [file ijms-23-04663-s001.zip › ijms-1639567supp/Supplementary Files Figures/Figure S2.png]

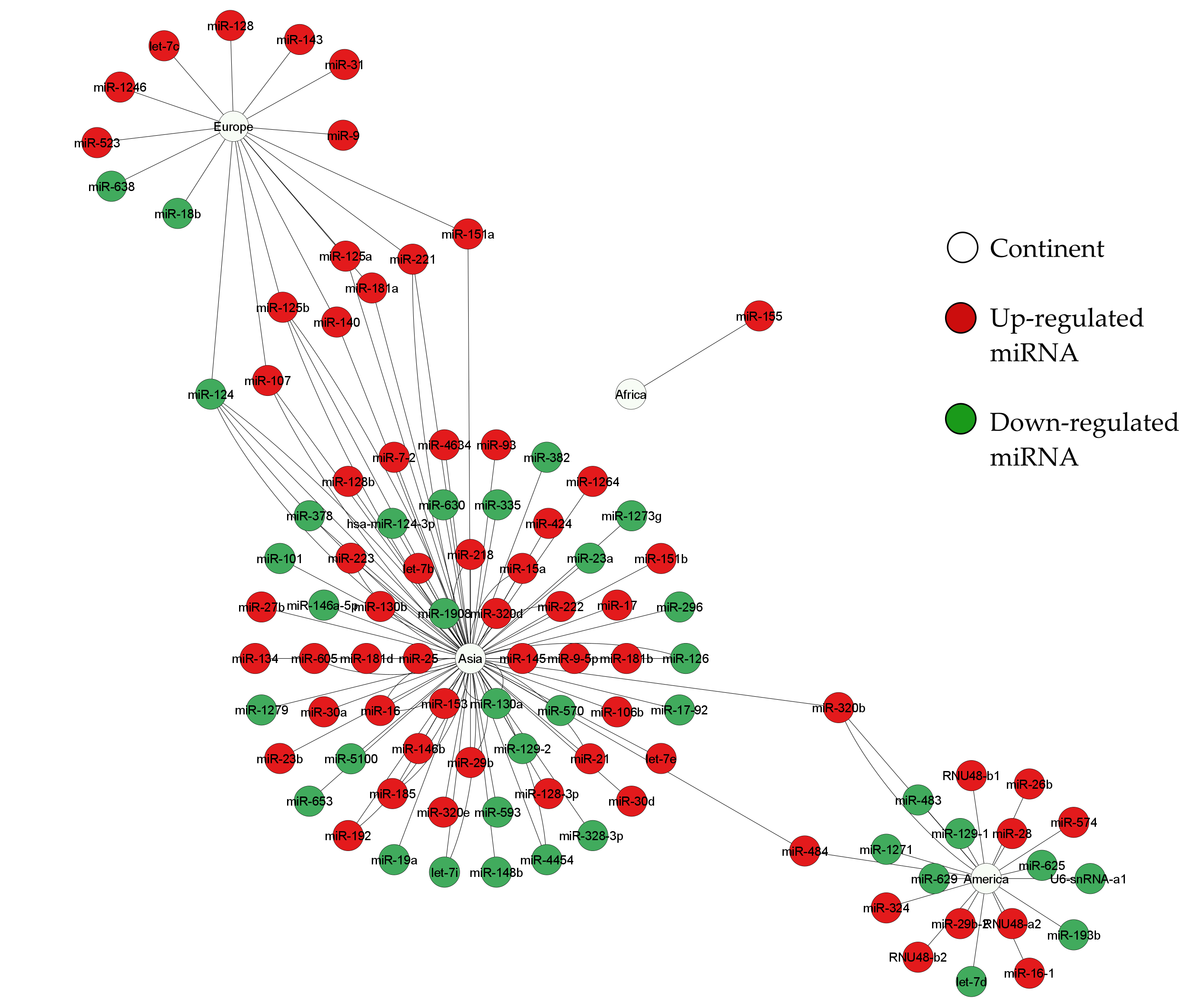

Supplement: Supplementary file 1 [file ijms-23-04663-s001.zip › ijms-1639567supp/Supplementary Files Figures/Figure S3.png]

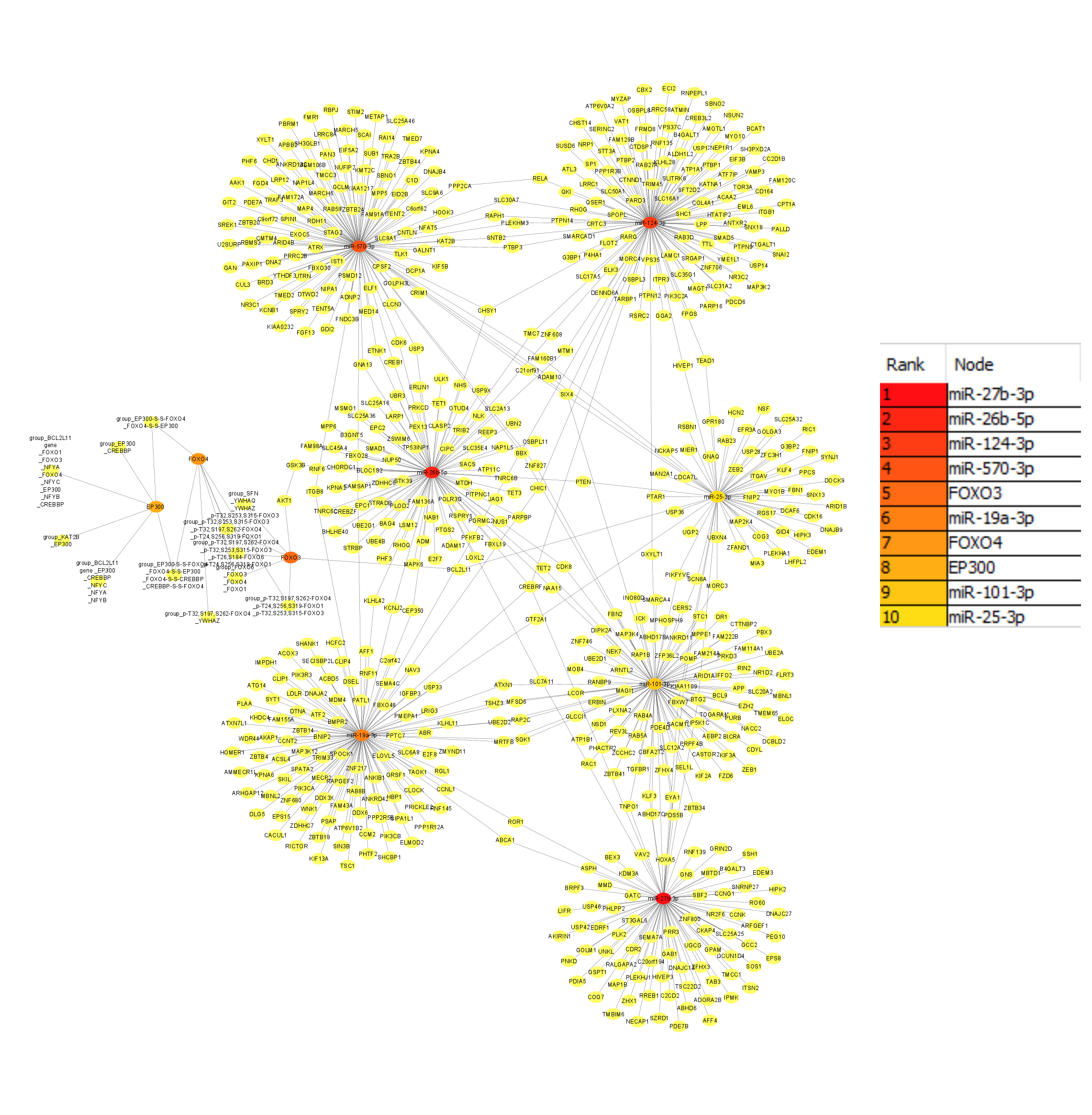

Supplement: Supplementary file 1 [file ijms-23-04663-s001.zip › ijms-1639567supp/Supplementary Files Figures/Figure S4-1.png]

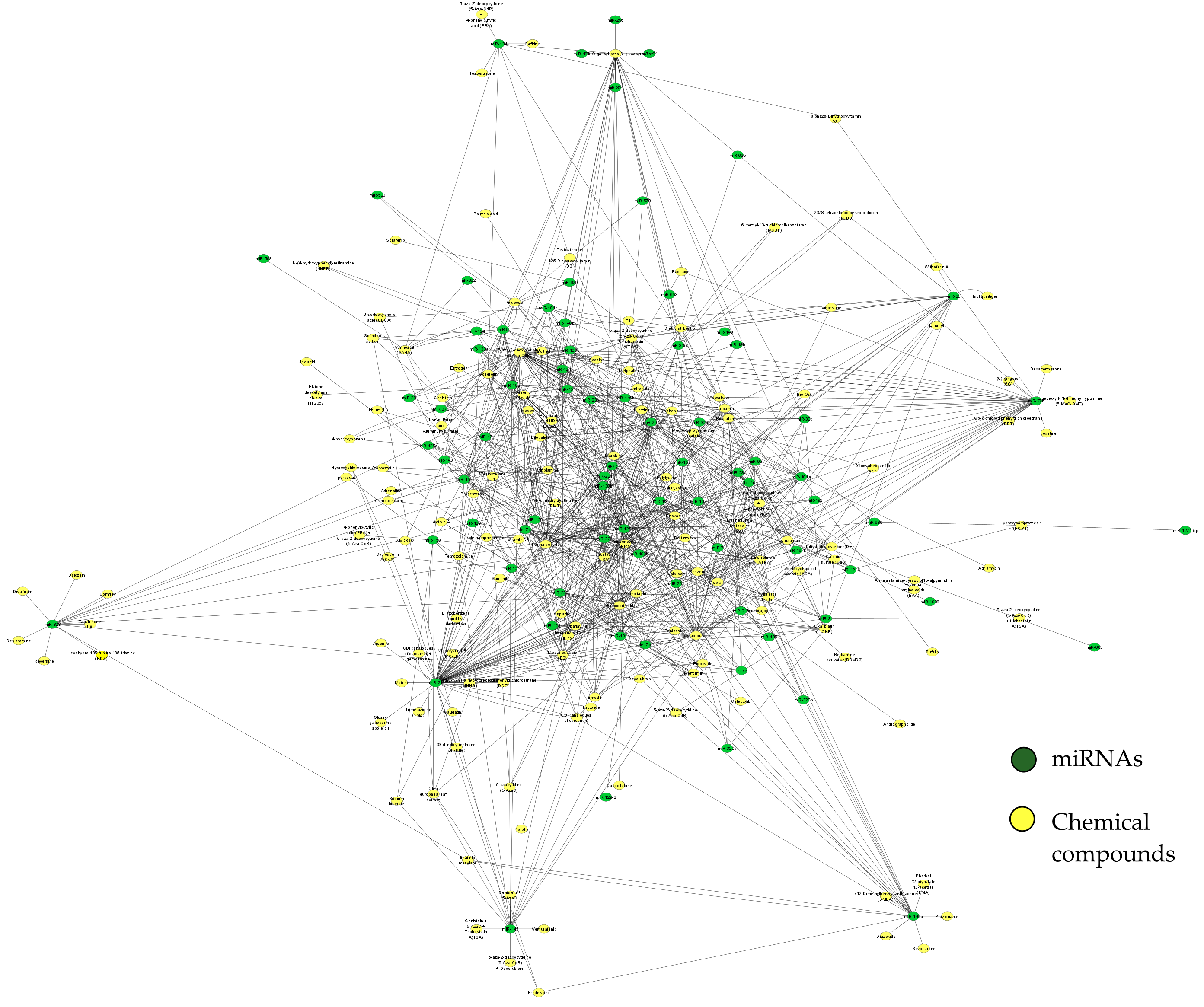

Supplement: Supplementary file 1 [file ijms-23-04663-s001.zip › ijms-1639567supp/Supplementary Files Figures/Figure S5.png]
